# Advancements in understanding the mechanisms of lung–kidney crosstalk

**DOI:** 10.1186/s40635-024-00672-1

**Published:** 2024-09-16

**Authors:** Renata de Souza Mendes, Pedro Leme Silva, Chiara Robba, Denise Battaglini, Miquéias Lopes-Pacheco, Celso Caruso-Neves, Patricia R. M. Rocco

**Affiliations:** 1 Department of Nephrology, State University of Rio de Janeiro, Rio de Janeiro, Brazil; 2 Department of Nephrology, Federal University of Rio de Janeiro, Rio de Janeiro, Brazil; 3 Laboratory of Pulmonary Investigation, Carlos Chagas Filho Institute of Biophysics, Federal University of Rio de Janeiro, Centro de Ciências da Saúde, Avenida Carlos Chagas Filho, 373, Bloco G-014, Ilha Do Fundão, Rio de Janeiro, RJ 21941-902 Brazil; 4 IRCCS Policlinico San Martino, Genoa, Italy; 5 Dipertimento di Scienze Chirurgiche Diagnostiche e Integrate, Policlinico San Martino, IRCCS Per l’Oncologia e Neuroscienze, Università degli Studi di Genova, Genoa, Italy; 6 Department of Pediatrics, Center for Cystic Fibrosis and Airway Disease Research, Emory University School of Medicine, Atlanta, GA USA; 7 Laboratory of Biochemistry and Cellular Biology, Carlos Chagas Filho Institute of Biophysics, Federal University of Rio de Janeiro, Rio de Janeiro, Brazil

**Keywords:** Acute kidney injury, Acute respiratory distress syndrome, Inflammation, Extracorporeal membrane oxygenation, Kidney, Lung

## Abstract

This narrative review delves into the intricate interplay between the lungs and the kidneys, with a focus on elucidating the pathogenesis of diseases influenced by immunological factors, acid–base regulation, and blood gas disturbances, as well as assessing the effects of various therapeutic modalities on these interactions. Key disorders, such as anti-glomerular basement membrane (anti-GBM) disease, the syndrome of inappropriate antidiuretic hormone secretion (SIADH), and Anti-neutrophil Cytoplasmic Antibodies (ANCA) associated vasculitis (AAV), are also examined to shed light on their underlying mechanisms. This review also explores the relationship between acute respiratory distress syndrome (ARDS) and acute kidney injury (AKI), emphasizing how inflammatory mediators can lead to systemic damage and impact multiple organs. In ARDS, fluid overload exacerbates pulmonary edema, while imbalances in blood volume, such as hypovolemia or hypervolemia, can precipitate renal dysfunction. The review highlights how mechanical ventilation strategies can compromise renal blood flow, trigger systemic inflammation, and induce hemodynamic and neurohormonal alterations, all contributing to lung and kidney damage. The impact of extracorporeal membrane oxygenation (ECMO) on lung–kidney interactions is evaluated, highlighting its role in severe respiratory failure and its renal implications. Emerging therapies, such as mesenchymal stem cells and extracellular vesicles, are discussed as promising avenues to mitigate organ damage and enhance outcomes in critically ill patients. Overall, this review offers a nuanced exploration of lung–kidney dynamics, bridging historical insights with contemporary perspectives. It underscores the clinical significance of these interactions in critically ill patients and advocates for integrated management approaches to optimize patient outcomes.

## Introduction

Inter-organ crosstalk represents a fundamental mechanism of bilateral communication, essential for maintaining physiological homeostasis through intricate cellular interactions, immune responses, and neurohormonal regulation [1]. However, under pathological conditions, this intricate interplay can precipitate or exacerbate primary injury in one or multiple organs [2].

Recent research has focused on the intricate interactions between lungs and kidneys, particularly in pulmonary–renal syndromes. These syndromes arise from various factors, including immunological triggers, such as anti-glomerular basement membrane (anti-GBM) antibody disease [3], the syndrome of inappropriate antidiuretic hormone secretion (SIADH) [4], and anti-neutrophil cytoplasmic antibody (ANCA)-associated vasculitis (AAV) [5]. Additionally, disturbances in fluid regulation, acid–base balance, and blood gas dynamics, such as hypoxemia or hypercapnia, can lead to tissue injury in both the respiratory and renal systems. [6].

Kidney disorders can instigate metabolic acidosis and fluid retention, activating the lung–buffer interaction and potentially fostering pulmonary damage by hindering the clearance of inflammatory mediators [7]. Moreover, alterations in renal hemodynamics and neurohormonal responses can further contribute to lung injury. Conversely, lung pathologies, particularly those requiring mechanical ventilation, can trigger systemic inflammatory responses that increase the risk of kidney injury [7].

Extracorporeal interventions, such as renal replacement therapy (RRT) and extracorporeal membrane oxygenation (ECMO), significantly influence lung–kidney crosstalk dynamics. While RRT can influence pulmonary function, ECMO may have adverse effects on renal function [8].

Emerging therapies, such as mesenchymal stem cells and extracellular vesicles (EVs), show promise in modulating lung–kidney interactions. EVs, key players in intercellular communication, may play a critical role in the pathogenesis of diseases affecting both organs [9, 10].

This narrative review seeks to elucidate the physiological interactions between lungs and kidneys, explore specific disorders, such as anti-GBM disease, SIADH, and AAV, and assess the impact of therapeutic modalities including mechanical ventilation, ECMO, and RRT. By deepening our understanding of these interactions, we aim to underscore their implications for overall body homeostasis and improve clinical outcomes for patients with related conditions.

### Physiologic interactions between lungs and kidneys

The lungs regulate blood pH by controlling CO₂ levels, adjusting breathing to manage these levels, which directly influences pH. The kidneys contribute to acid–base balance by excreting hydrogen ions, reabsorbing, and producing bicarbonate. Additionally, the lungs affect fluid balance through respiratory water loss, which can be exacerbated by hyperventilation. The kidneys maintain fluid balance by filtering blood, reabsorbing essentials, and excreting excess water and electrolytes [11]. The lungs also produce ACE, influencing blood pressure, while the kidneys release hormones like erythropoietin and renin, critical for blood pressure regulation and red blood cell production [12].

### Classic interactions between kidneys and lungs

Classic interactions between kidneys and lungs encompass various factors, including immunologic conditions like anti-GBM disease, disturbances in water regulation seen in SIADH, AAV, as well as disruptions in acid–base balance and blood gas levels.

### Anti-glomerular basement membrane disease

Anti-GBM disease exemplifies the severe impact of immune overactivation on both respiratory and renal systems. This rare condition is marked by antibodies, particularly IgG1 and IgG3, targeting antigens in the basement membrane, including the alpha-3 domain of type IV collagen, peroxidasin, and laminin-521. These antibodies attack glomerular capillaries in the kidneys and alveolar capillaries in the lungs, leading to crescent glomerulonephritis and pulmonary hemorrhage in about 50% of cases [3].

Genetic factors, notably specific HLA types, influence susceptibility to anti-GBM disease. HLA-DR1 and DR7 offer some protection, while HLA-DR15 and DR4 increase susceptibility by exposing epitopes that lead to immune dysregulation [13]. Epigenetic and environmental factors, such as pulmonary infections (including COVID-19), smoking, exposure to hydrocarbon solvents, and lung cancer, further elevate the risk by triggering or worsening the immune response [14].

Understanding the complex interplay between genetic susceptibility, environmental triggers, and immune dysregulation is crucial for managing and treating anti-GBM disease. Further research into these areas may provide insights into the mechanisms of the disease and potential therapeutic targets.

### Syndrome of inappropriate antidiuretic hormone secretion

Vasopressin, or antidiuretic hormone (ADH), is secreted by the hypothalamus and regulates osmotic balance, blood pressure, and kidney function. Under normal conditions, vasopressin levels rise in response to elevated plasma osmolarity or reduced blood volume, prompting water reabsorption in the kidneys to maintain homeostasis [15]. However, in conditions like pneumonia, asthma, atelectasis, respiratory failure, mechanical ventilation, neoplasia, and pneumothorax, vasopressin production can increase inappropriately, independent of osmolarity or blood volume, leading to imbalances in fluid regulation [15].

### Anti-neutrophil cytoplasmic antibodies-associated vasculitis

Anti-neutrophil cytoplasmic antibody-associated vasculitis involves both lungs and kidneys, leading to significant morbidity and mortality. The pathogenesis of AAV is driven by a complex interplay of immune responses, endothelial damage, and inflammation. In AAV, the immune system produces ANCAs that target proteins like myeloperoxidase or proteinase 3 on neutrophils. When ANCAs bind to these proteins, they activate neutrophils, which then release reactive oxygen species and proteolytic enzymes, causing tissue damage. This process damages endothelial cells, increases vascular permeability, and triggers inflammation, marked by the release of pro-inflammatory cytokines, such as tumor necrosis factor (TNF)-α, interleukin (IL)-1, and IL-6. This leads to vasculitis, granuloma formation, and further tissue injury in the lungs and kidneys. The complement system, especially the alternative pathway, also plays a critical role in mediating inflammation and tissue injury in AAV [15].

In the lungs, AAV can cause diffuse alveolar hemorrhage due to damage to pulmonary capillaries, while chronic inflammation may lead to interstitial lung disease with fibrosis and reduced lung function. In the kidneys, similar immune mechanisms result in glomerulonephritis and crescent glomerulonephritis, leading to progressive renal dysfunction.

### Disruption in acid–base balance

Acid–base balance is tightly regulated by the lungs and kidneys to maintain plasma pH within the narrow range of 7.35–7.45, essential for optimal cellular function. The lungs manage pH by eliminating carbon dioxide, while the kidneys maintain balance by reabsorbing bicarbonate and generating new bicarbonate through titratable acidity and ammonia excretion. This interaction becomes critical in patients with severe lung disease, especially those on mechanical ventilation. Improper ventilator settings can exacerbate acid–base imbalances, placing additional strain on the kidneys [16].

Bicarbonate (HCO_3_^−^) and arterial partial pressure of carbon dioxide (PaCO_2_) are key in this regulation, with bicarbonate directly and PaCO_2_ inversely affecting pH. In chronic conditions like chronic obstructive pulmonary disease (COPD), where CO_2_ retention is common, the kidneys' ability to regenerate bicarbonate is vital. However, patients with renal dysfunction or advanced chronic kidney disease may struggle to maintain pH balance, leading to respiratory acidemia and increased mortality risk [17].

COPD is often accompanied by malnutrition, muscle wasting, and renal issues, which further compromise acid–base regulation. Diuretic use in COPD management can also impact renal function and acid–base balance. Effective management of chronic hypercapnia in COPD requires a comprehensive approach, including optimizing ventilator settings, monitoring medications, and ensuring adequate nutritional support [18].

In summary, lungs and kidneys work together to maintain pH balance, and dysfunction in either system can severely disrupt this delicate equilibrium, particularly in patients with chronic lung disease [19].

### Blood gas disturbances

Renal blood flow is regulated by factors like the myogenic reflex and tubuloglomerular feedback [20], making kidneys highly sensitive to hypoxic injuries due to their high oxygen demand per gram of tissue [21]. This demand is influenced by several factors, including glomerular–tubular balance, sodium reabsorption, and metabolic activity. In systemic hypercapnia, the increased need for bicarbonate reabsorption and regeneration affects the GFR [22]. Neurohormonal regulators, including angiotensin II, nitric oxide, and adrenergic nerves, are crucial for balancing oxygen supply and demand. If vasodilatory responses fail, compensatory mechanisms may not suffice, potentially leading to renal injury [16].

Lung diseases that alter blood gas values, such as hypoxemia or hypercapnia, can significantly impact renal hemodynamics. Acute hypercapnia reduces renal blood flow, a condition more pronounced in patients with exacerbated COPD [23, 24]. This reduction is caused by both direct renal vasoconstriction and indirect mechanisms, including systemic vasodilation and baroreceptor inactivation, leading to norepinephrine release and further reduced renal blood flow [23, 24]. Neurogenic mechanisms play a key role in this process, as demonstrated by a study showing that denervated kidneys do not exhibit the same blood flow reduction [25].

Hypercapnia can also increase pulmonary vascular resistance, potentially leading to right ventricular dysfunction [26]. Chronic respiratory disorders often result in fluid retention, pulmonary edema, and congestion, associated with reduced renal blood flow and impaired sodium excretion [24].

Hypoxemia also affects renal blood flow, possibly through a reflex mechanism involving sympathetic nerves and chemoreceptor stimulation. The role of nitric oxide (NO) in this response is suggested, as COPD patients often show a lack of response to L-arginine, indicating potential deficiencies in NO-mediated vasodilation [27].

In summary, disruptions in blood gas levels due to lung disease can profoundly impact renal hemodynamics, with hypercapnia and hypoxemia leading to reduced renal blood flow and potential renal injury.

### The role of ARDS in the development of AKI

Acute kidney injury occurs in approximately 30% of ARDS patients, associating with increased mortality rates and prolonged hospital stays [28]. Nearly half of these individuals also require RRT [29]. While ARDS primarily impairs kidney function through changes in pulmonary circulation, right ventricular function, and fluid balance, factors like mechanical ventilation, hypoxemia, hypercapnia, and systemic inflammation also contribute to kidney dysfunction [30].

Fluid overload is common in ARDS patients and can raise pressure in the right heart chambers, worsening venous congestion and pulmonary hypertension, particularly in cases of severe hypoxia [31]. This increased systemic venous pressure leads to elevated renal interstitial and intratubular pressures, reducing renal perfusion and oxygen delivery, ultimately causing AKI. Additionally, fluid overload in the pulmonary microcirculation can exacerbate pulmonary edema and impair gas exchange [31]. Although a conservative fluid resuscitation strategy may reduce ICU stay, striking a balance between avoiding hypovolemia and ensuring adequate tissue perfusion remains challenging [32]. The type of fluid administered can also influence the severity of hydrostatic and inflammatory pressures in ARDS, though the clinical efficacy of hypo-osmolar albumin, suggested by animal studies, remains uncertain [33, 34].

Hypoxemia and hypercapnia further affect renal circulation by inducing pulmonary arterial vasoconstriction and hypertension, leading to venous congestion. Severe pulmonary hypertension can result in acute right ventricular dysfunction (cor pulmonale), which, if untreated, increases the risk of AKI by elevating interstitial and intratubular pressures and reducing renal perfusion [31].

The use of high positive end-expiratory pressure (PEEP) in mechanical ventilation also increases the risk of AKI in ARDS patients by diminishing renal perfusion due to its hemodynamic effects [29].

### The impact of AKI on the lungs

Acute kidney injury triggers significant damage to the tubular epithelium, leading to cell death and the release of inflammatory mediators and oxidative stress, which can adversely affect distant organs, including the lungs [19]. The lungs, with their extensive capillary network, are particularly vulnerable to the systemic effects of AKI, such as increased cytokine production, disrupted nitric oxide metabolism, leukocyte infiltration, and increased pulmonary vascular permeability. Additionally, AKI reduces the expression of critical components like epithelial sodium channels, sodium–potassium adenosine triphosphatase (ATPase), and aquaporin 5, all of which are vital for efficient alveolar fluid clearance.

Experimental studies have highlighted other factors contributing to this pathophysiology, including the systemic release of damage-associated molecular patterns (DAMPs) from necrotic renal cells, caspase-dependent apoptotic pulmonary cell death mediated by TNF-α receptors, and microvascular dysfunction of the alveolar-capillary barrier [35, 36]. The complex interplay between the lung and kidney is exemplified by the use of furosemide, a diuretic that inhibits sodium–potassium-chloride cotransporters. This action not only prevents the secretion of alveolar fluids, alleviating pulmonary edema, but also demonstrates the intricate connection between these organs, as the drug's effects on the lungs occur even before increased diuresis is observed [37].

### Therapeutic interventions

The complex interplay between kidney and lung highlights the need for integrated therapeutic strategies, particularly in conditions like AKI and ARDS. Dysfunction in one organ can precipitate pathology in the other, driven by systemic inflammation, hemodynamic changes, and shared pathogenic pathways. Understanding this bidirectional crosstalk is crucial for improving patient outcomes.

### Mechanical ventilation

#### The impact of mechanical ventilation on renal function through hemodynamic impairment

While mechanical ventilation is essential for critically ill patients, it can adversely affect renal function, particularly through hemodynamic impairment [18, 38]. Positive pressure ventilation, unlike spontaneous breathing, maintains pressure throughout the respiratory cycle, which can reduce GFR by influencing airway pressure, tidal volume, and patient volume status [39]. Elevated PEEP has been linked to decreased renal blood flow and GFR. On the other hand, assisted breathing, which activates the diaphragm, can improve renal function and electrolyte balance, reflecting the physiological differences from controlled ventilation [40].

Increased intrathoracic pressure from mechanical ventilation can impair right ventricular function and elevate pulmonary resistance, leading to renal congestion [41]. This congestion arises from increased central venous pressure, reduced arteriovenous gradient within the kidney, and diminished renal blood flow, ultimately causing renal venous hypertension and potential parenchymal congestion. These effects can lead to local hypoxia due to reduced capillary blood flow, exacerbating kidney dysfunction [42].

Intra-abdominal pressure, influenced by diaphragmatic contraction and abdominal compartment characteristics, also affects renal perfusion [43]. Mechanical ventilation can exacerbate this pressure, compromising renal microvascular blood flow, depending on airway and baseline abdominal pressures. The resulting renal edema can perpetuate a cycle of intra-abdominal pressure elevation, further impairing kidney function.

A restrictive fluid strategy is often employed in managing critically ill patients to improve lung injury outcomes. However, in hypovolemic patients, particularly those on positive pressure ventilation, this can deteriorate renal perfusion, increasing the risk of AKI [44].

#### The impact of mechanical ventilation on renal function through neurohormonal regulation

**Fig. 1:**
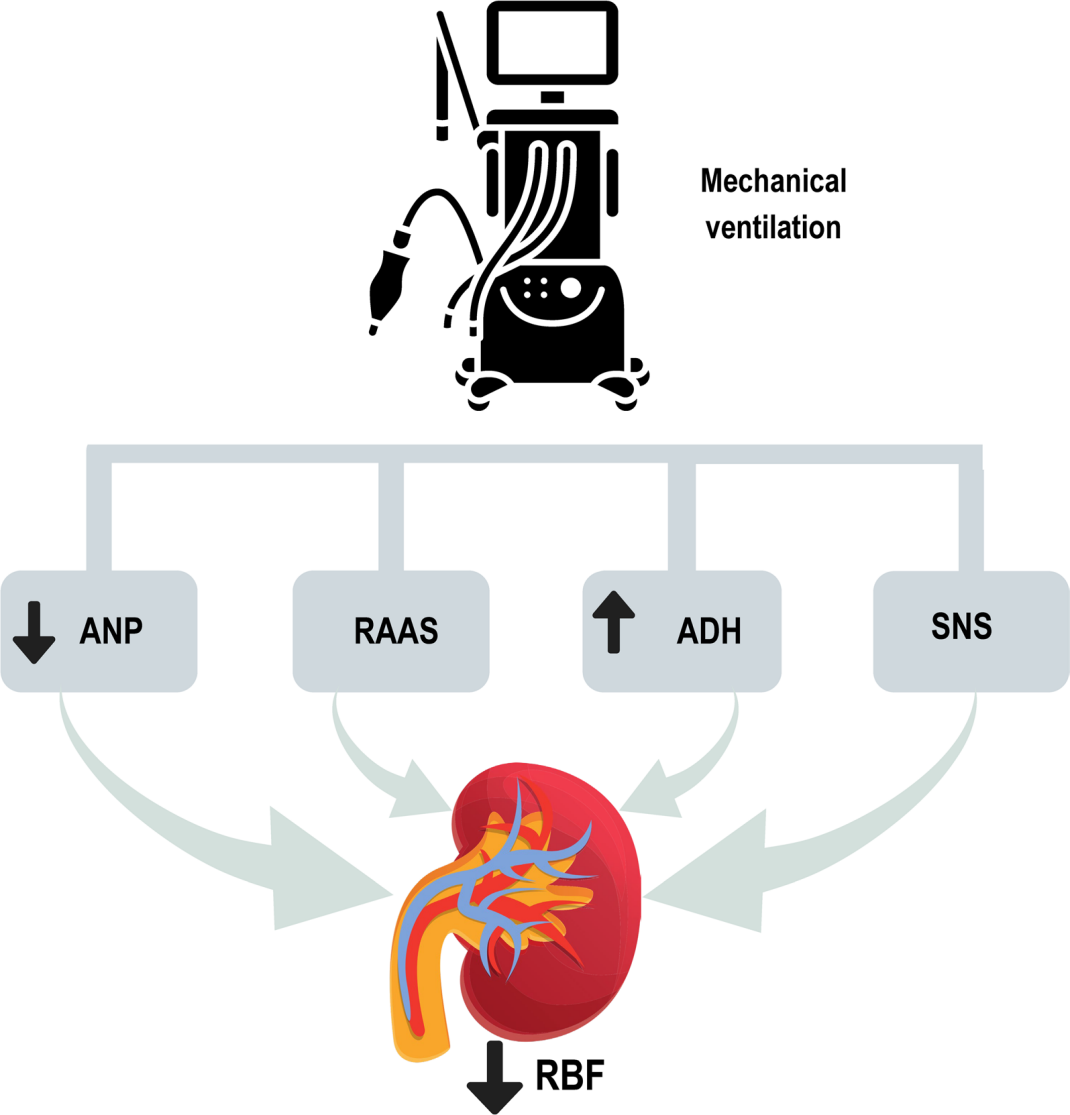
Mechanical ventilation can affect renal blood flow through several interconnected mechanisms. ADH, antidiuretic hormone (also known as vasopressin); *ANP* atrial natriuretic peptide, *RAAS* renin–angiotensin–aldosterone system, *RBF* renal blood flow, *SNS* sympathetic nervous system

Mechanical ventilation impacts renal function through neurohormonal regulation. Atrial natriuretic peptide (ANP) responds to fluid balance changes by promoting fluid excretion and influencing renal blood flow. The renin–angiotensin–aldosterone system (RAAS), activated by reduced renal perfusion, produces angiotensin II and aldosterone, leading to vasoconstriction and fluid retention, which alter renal blood flow. Elevated antidiuretic hormone (ADH) levels promote water reabsorption but may be less effective if mechanical ventilation compromises cardiac output. Sympathetic nervous system (SNS) activation also causes renal vasoconstriction, further impacting blood flow. These interactions illustrate the intricate physiological relationship between mechanical ventilation and renal perfusion [45–47] (Fig. 1).

#### The impact of mechanical ventilation on renal function through the release of biomarkers

Non-protective mechanical ventilation strategies can lead to the release of pro-inflammatory mediators (e.g., TNF-α, IL-1β, IL-6, and IL-8) from alveolar cells, making the kidneys particularly vulnerable to injury [48, 49]. This can result in renal epithelial cell apoptosis, changes in plasma creatinine levels, and alterations in vasoactive mediators, coagulation, and fibrinolysis, alongside factors like vascular endothelial growth factor [50–52].

The RAAS, activated by mechanical ventilation, plays a crucial role in developing acute lung injury and ARDS [53]. While ACE catalyzes the conversion of angiotensin I to angiotensin II, ACE2 counteracts angiotensin II, serving as a negative regulator of the system. The significance of ACE2 became evident during the COVID-19 pandemic [54], where SARS-CoV-2 used ACE2 as its primary receptor to infect lung cells, disrupting the RAAS pathway and leading to uncontrolled angiotensin II elevation, associated with severe disease forms.

In chronic kidney disease (CKD), RAAS inhibitors have effectively slowed disease progression [55, 56], highlighting the RAAS's role in transitioning from AKI to CKD. Recent studies show that blocking renin–angiotensin signaling can reduce CKD development and associated mortality following AKI [57]. Kidney dysfunction can worsen lung edema and impair lung function, with elevated levels of ACE, angiotensin II, and AT1R playing a key role in this process [53].

### Extracorporeal therapies

ECMO and continuous RRT (CRRT) provide significant benefits in managing severe ARDS and AKI that are refractory to conventional treatments. ECMO can provide respiratory support while mitigating the deleterious effects of mechanical ventilation on the kidneys, and CRRT can aid in fluid balance and the removal of inflammatory mediators, supporting lung function [58].

### Crosstalk between renal support therapies and extracorporeal membrane oxygenation

The incidence of AKI in patients requiring ECMO exceeds 70% and approximately 50% also need renal replacement therapy [29]. Patients with AKI, compared with those without, often experience prolonged mechanical ventilation, extended ECMO support, and higher mortality rates [30].

The primary mechanisms attributed to renal injury during and after ECMO include hypoperfusion, prolonged hypoxemia, ischemia–reperfusion injury after initiation of ECMO, monophasic flow in veno-arterial ECMO, and improper cannula positioning leading to venous or arterial obstruction or platelet disruption resulting in fat embolism [59]. Hemolysis is also a potential complication during extracorporeal therapy, yielding erythrocyte fragmentation due to mechanical stress within the extracorporeal system, air–fluid interface, and excessive negative pressure. This can occur, for instance, with poorly flowing cannulas or in hypovolemic patients [59].

Exposure of blood to the extracorporeal circuit can induce a state of hypercoagulability, resulting in microvascular dysfunction and endothelial damage, ultimately leading to reduced tissue oxygen supply [21]. Along with endothelial dysfunction and consumption of coagulation factors, the use of anticoagulants required for surgeries increases the risk of bleeding, which can affect the outcomes of these patients [21].

Patients undergoing extracorporeal therapies are more sensitive to extremes in fluid balance because both fluid overload and dehydration can impair cardiac, renal, and pulmonary function. Initiation of ECMO can lead to hemodynamic fluctuations due to membrane biocompatibility, leading to AKI through mechanisms of ischemia–reperfusion injury. Furthermore, previously undiagnosed hypovolemia at the time of cannulation can exacerbate renal injury if not promptly addressed.

Similarly, fluid accumulation can have deleterious effects on critically ill patients, potentially accumulating in the interstitium, which exacerbates oxygen impairment, and further reduces glomerular filtration due to congestive syndrome. Veno-arterial ECMO affects hemodynamics differently from veno-venous ECMO. Veno-venous ECMO maintains the pulsatile flow of cardiac output with less impact on renal blood flow. In veno-arterial ECMO, there is a mix of pulsatile flow generated by the heart and non-pulsatile flow generated by ECMO propulsion. Furthermore, the flow in veno-arterial ECMO bypasses the lungs, increasing the risk of embolization, which, combined with the non-pulsatile flow, results in a higher risk of vascular occlusion. Other adverse effects of this laminar flow include changes in endothelial integrity, thus favoring the formation of edema [60], increased RAAS activity, and consequently, reduced renal blood flow [61].

However, when veno-venous ECMO (VV-ECMO) is used to control refractory hypoxemia and/or hypercapnia, it can help mitigate some of the pathophysiological mechanisms contributing to AKI. By improving oxygenation and reducing carbon dioxide levels, VV-ECMO can enhance renal perfusion and alleviate *cor pulmonale*. Additionally, it enables the use of lung-protective mechanical ventilation strategies.

Therefore, controlling the hemodynamic and mechanical effects of veno-venous ECMO can have a benefit of ECMO in controlling the causative factors of AKI in this population [62]. Hence, renal function must be monitored in patients requiring ECMO because they are at a higher risk of AKI and, consequently, a higher risk of mortality [21].

#### Integration of ECMO and CRRT

Integration of ECMO and CRRT poses a significant challenge in patients requiring both therapies simultaneously, necessitating careful consideration to prevent potential complications. The primary concerns revolve around bleeding, embolism, coagulation, and hemolysis.

Two integration approaches exist: parallel and series connections, each with its own set of advantages and drawbacks. In parallel connection, the procedures operate independently with separate vascular access points, mitigating risks of embolism, coagulation, and hemolysis. However, the need for additional vascular access raises concerns in critically ill patients already requiring multiple access points and anticoagulation, potentially increasing the risk of iatrogenic complications.

**Fig. 2:**
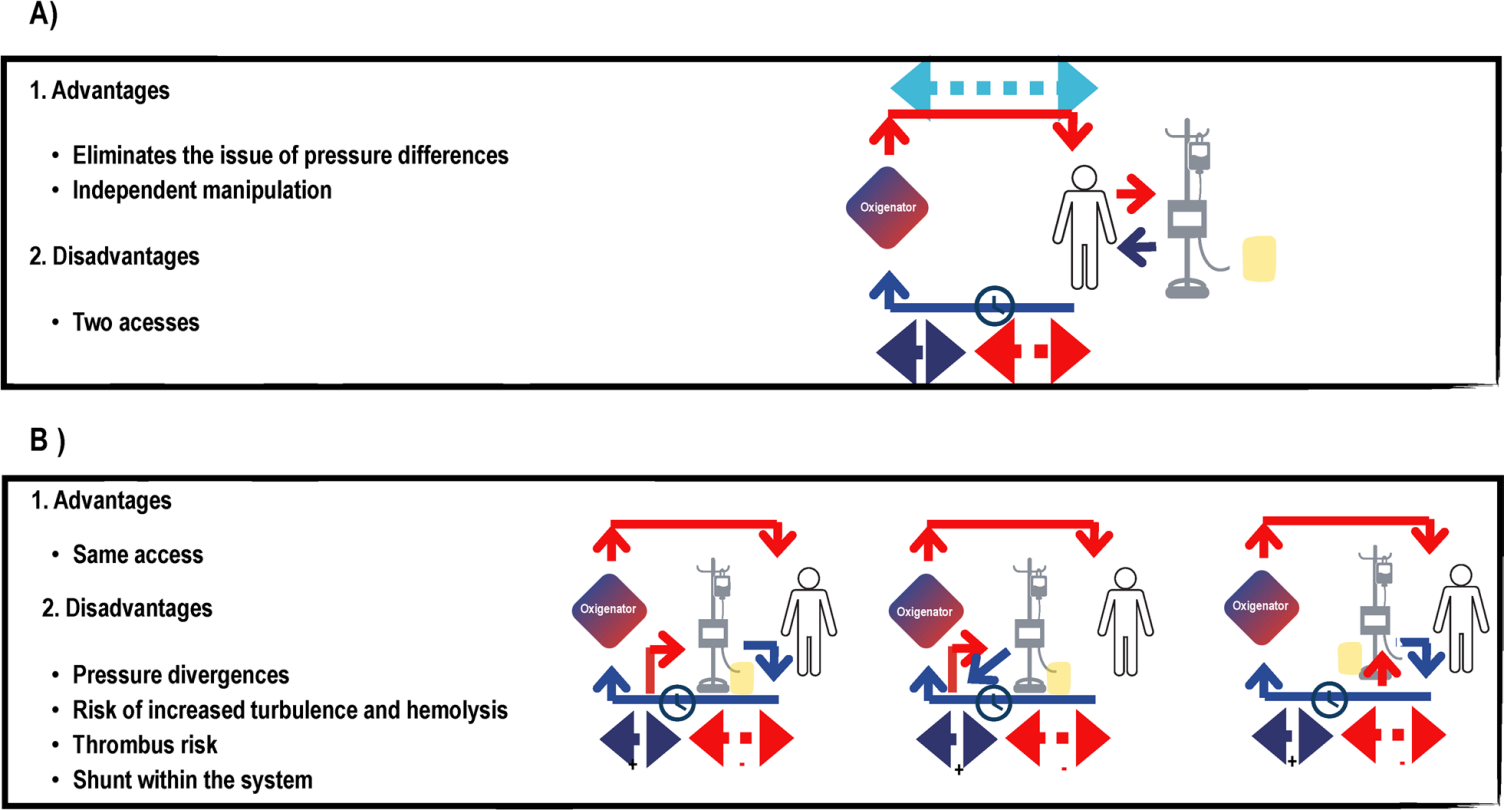
Types of extracorporeal membrane oxygenation and continuous renal replacement therapy connection: advantages and disadvantages. **A** Parallel connection; **B** series connection

One major challenge in integration is reconciling the significantly different flows and pressures required by each system. ECMO typically necessitates blood flows of 70 to 80 ml/kg/min for adequate oxygenation, while CRRT operates at much lower blood flows of 100 to 150 ml/min. CRRT equipment is designed to function safely at lower pressures than those encountered in the ECMO circuit. In a series connection, the challenge lies in coupling the CRRT machine to an ECMO site with pressure compatible for CRRT operation without exacerbating risks of embolization and coagulation. Safe connection sites include the pre-pump territory of ECMO (negative pressure) and the zone between the ECMO blood pump and the oxygenator, while the post-oxygenator territory is discouraged due to the increased risk of embolization. Pressure disparities between systems elevate the risk of turbulence, hemolysis, frequent interruptions in the CRRT system, and subsequent circuit coagulation **(**Fig. 2**)**.

Despite these challenges, series connection offers the advantage of requiring access only for ECMO, minimizing the risks associated with multiple punctures and anticoagulation. Therefore, the optimal integration method depends on the patient's clinical condition and the expertise of the healthcare team.

### Mesenchymal stem cell therapy and the crosstalk between lung and kidneys

Mesenchymal stem cells (MSCs) have garnered considerable attention for their regenerative potential and their role in mediating organ repair. Recent research underscores their significance in the context of inter-organ crosstalk, particularly between the kidneys [63] and lungs [64]. MSC therapeutic efficacy is largely attributed to their immunomodulatory properties, ability to promote tissue repair, and capacity to secrete EVs that carry cytokines, growth factors, and genetic material capable of influencing distant tissues. MSCs have been shown to attenuate systemic inflammation, promote tissue repair and regeneration, and may also impact hemodynamic and neurohormonal regulation by modulating the systemic response to organ injury in ARDS [65] and AKI [66]. The application of MSCs therapy in clinical settings holds significant promise for treating diseases involving lung–kidney interactions.

### The role of extracellular vesicles in the crosstalk between lung and kidneys

EVs are membrane-bound structures devoid of a nucleus, facilitating vital intercellular communication by transferring diverse biological cargoes, such as proteins, messenger ribonucleic acid (mRNA), and microRNA, between different cell types. While robust evidence is still evolving, the biological plausibility of EVs in mediating organ communication in response to damage is evident [10, 58].

EVs are likely to participate in exchanging harmful mediators between the kidney and lungs through endocrine actions, while also engaging in protective measures to mitigate damage, as observed in models of trauma and hemorrhagic shock [67]. Additionally, EVs hold therapeutic promise in modulating various pathogenic mechanisms underlying organ crosstalk, including endothelial dysfunction, thrombo-inflammation, oxidative stress, and macrophage phenotypic changes [9]. The interplay between EVs from renal tubular epithelial cells and neighboring macrophages, as well as between lung epithelial cells and alveolar macrophages, constitutes a significant mechanism underlying injury in AKI and ARDS, respectively. The potential to attenuate this process using mesenchymal stromal cell-derived EVs or employing epithelial-derived or endothelial-derived EVs to shield endothelial cells from lipopolysaccharide (LPS)-induced permeability holds promise as an etiopathogenetic treatment approach [10, 68].

Moreover, the immunomodulatory properties of EVs extend beyond local damage mechanisms, potentially influencing distant organ crosstalk and inflammatory dysregulation, characteristic of conditions such as sepsis. Future clinical studies should focus on essential aspects that facilitate the broader implementation of EV-based therapy [10, 68].

### Other therapies

#### Anti-inflammatory therapies

Targeting the inflammatory cascade is pivotal in mitigating the crosstalk between AKI and ARDS. Therapies, such as corticosteroids, cytokine inhibitors, and novel anti-inflammatory agents, can potentially reduce systemic inflammation and protect both renal and pulmonary function [69].

#### Biomarker-Guided Therapies

The identification and the utilization of biomarkers specific to kidney–lung crosstalk can facilitate early diagnosis and tailored interventions. Biomarkers, such as neutrophil gelatinase-associated lipocalin (NGAL) and interleukin-6 (IL-6), may guide therapeutic decisions and monitor response to treatment [70].

## New perspectives and future directions

The evolving understanding of kidney–lung crosstalk opens new avenues for research and therapeutic innovation. EVs, carrying proteins, lipids, and nucleic acids, could serve as novel therapeutic targets or biomarkers for early detection and intervention in AKI and ARDS.

Regenerative medicine also holds potential, with MSCs offering anti-inflammatory and regenerative properties that could be harnessed to repair and protect both renal and pulmonary tissues. Clinical trials are needed to establish optimal dosing regimens, delivery methods, and patient selection criteria. Moreover, ongoing research into the mechanisms underlying MSC-mediated effects on organ interactions will be critical for refining therapeutic approaches and maximizing their efficacy.

## Conclusions

The crosstalk between the lungs and kidneys represents a dynamic and bidirectional communication system essential for maintaining physiological homeostasis. This intricate interplay involves a variety of mechanisms and pathways that enable coordinated responses to changes in the internal environment and external stressors. One aspect of lung–kidney crosstalk involves immunological factors, fluid and electrolyte balance, hemodynamic interactions, neurohormonal responses, inflammatory mediators (key signaling molecules in lung–kidney crosstalk), and therapeutic interventions. Understanding the complexities of lung–kidney crosstalk is crucial for optimizing the management of patients with respiratory and renal conditions. This holistic perspective underscores the interconnectedness of these vital organ systems and highlights the importance of integrated therapeutic approaches in critical care medicine.

## Take-home message

Understanding the lung–kidney interplay is essential in managing critically ill patients. This review highlights the relationship between ARDS and AKI, where inflammatory mediators contribute to organ damage.

Renal replacement therapy (RRT) can reduce pulmonary edema but carries risks of hemodynamic instability, inflammation, and infection. AKI is common in patients requiring ECMO, leading to prolonged mechanical ventilation and higher mortality rates.

## Tweet

Exploring the intricate interplay between lungs & kidneys, and their essential roles in maintaining the body's homeostasis. #Health #Medicine #LungKidneycrosstalk.

## Data Availability

Not applicable.

## Abbreviations

ADH: Antidiuretic hormone
AKI: Acute kidney injury
Anti-GBM: Anti-glomerular basement membrane
ARDS: Acute respiratory distress syndrome
CKD: Chronic kidney disease
COPD: Chronic obstructive pulmonary disease;
CRRT: Continuous renal replacement therapy
ECMO: Extracorporeal membrane oxygenation
HLA: Human leukocyte antigen
ICU: Intensive care unit
NO: Nitric oxide
PaCO2: Arterial partial pressure of carbon dioxide
PEEP: Positive end-expiratory pressure
RAAS: Renin–angiotensin–aldosterone system
SIADH: Syndrome of inappropriate antidiuretic hormone secretion
